# Corticosterone Levels and Glucocorticoid Receptor Gene Expression in High Drinking in the Dark Mice and Their Heterogeneous Stock (HS/NPT) Founder Line

**DOI:** 10.3389/fnbeh.2022.821859

**Published:** 2022-05-12

**Authors:** Antonia M. Savarese, Kolter B. Grigsby, Bryan E. Jensen, Marissa B. Borrego, Deborah A. Finn, John C. Crabbe, Angela R. Ozburn

**Affiliations:** ^1^Portland Alcohol Research Center, Department of Behavioral Neuroscience, Oregon Health & Science University, Portland, OR, United States; ^2^VA Portland Health Care System, Portland, OR, United States

**Keywords:** ethanol, binge drinking, HDID, glucocorticoid, FKBP5

## Abstract

The High Drinking in the Dark (HDID-1) line of mice has been selectively bred for achieving high blood alcohol levels (BALs) in the Drinking in the Dark task, a model of binge-like drinking. Recently, we determined that glucocorticoid receptor (GR) antagonism with either mifepristone or CORT113176 (a selective GR antagonist) reduced binge-like ethanol intake in the HDID-1 mice, but not in their founder line, HS/NPT. Here, we examined whether the selection process may have altered glucocorticoid functioning by measuring (1) plasma corticosterone levels and (2) expression of the genes encoding GR (*Nr3c1*) and two of its chaperone proteins FKBP51 and FKBP52 (*Fkbp5* and *Fkbp4*) in the brains (nucleus accumbens, NAc) of HDID-1 and HS/NPT mice. We observed no genotype differences in baseline circulating corticosterone levels. However, HDID-1 mice exhibited a greater stimulated peak corticosterone response to an IP injection (of either ethanol or saline) relative to their founder line. We further observed reduced basal expression of *Fkbp4* and *Nr3c1* in the NAc of HDID-1 mice relative to HS/NPT mice. Finally, HDID-1 mice exhibited reduced *Fkbp5* expression in the NAc relative to HS/NPT mice following an injection of 2 g/kg ethanol. Together, these data suggest that selective breeding for high BALs may have altered stress signaling in the HDID-1 mice, which may contribute to the observed selective efficacy of GR antagonism in reducing binge-like ethanol intake in HDID-1, but not HS/NPT mice. These data have important implications for the role that stress signaling plays in the genetic risk for binge drinking.

## Introduction

Stress is a critical factor in alcohol use and misuse and has been shown to contribute to the development of alcohol use disorders (AUDs) as well as to the risk of relapse in individuals with an AUD (Blaine and Sinha, 2017). Acute alcohol activates the hypothalamic-pituitary-adrenal (HPA) axis, the body’s main neuroendocrine stress response, and chronic alcohol use can cause dysregulation of the HPA axis (Richardson et al., 2008). HPA activation culminates in the release of glucocorticoids (cortisol in humans and corticosterone in rodents) from the adrenal glands. Individuals who engage in binge/heavy drinking and those with an AUD often exhibit higher basal cortisol levels and a blunted cortisol response to stress or alcohol exposure (Sinha et al., 2009; Blaine et al., 2016). These effects can also be observed in rodents and non-human primates (Cippitelli et al., 2014; Jimenez and Grant, 2017; Somkuwar et al., 2017). This altered glucocorticoid output with chronic alcohol use is thought to contribute to craving for alcohol and promote further intake and relapse (Junghanns et al., 2005; Blaine et al., 2019).

As glucocorticoid levels rise, they bind to glucocorticoid receptors (GR) in the hypothalamus to terminate further glucocorticoid release. However, GR is also located throughout the brain, and activation of GR in extrahypothalamic brain regions may be particularly important for alcohol use (Edwards et al., 2015). Chronic alcohol use leads to dysregulated GR expression in extrahypothalamic brain regions that changes dynamically across the acute withdrawal and protracted abstinence periods, although the direction of these effects is not always consistent and is likely brain-region-specific. In rats made dependent on alcohol via exposure to chronic intermittent ethanol (CIE) vapor, elevated GR during the acute withdrawal period and reduced GR during protracted abstinence have been observed in the medial prefrontal cortex (mPFC) (Somkuwar et al., 2017), a brain region thought to be critical for alcohol relapse (Lu and Richardson, 2014). In contrast, in brain regions involved in stress and reward processing such as the nucleus accumbens (NAc, a major component of the ventral striatum) and the central nucleus of the amygdala, the reverse is seen: GR expression patterns in rats exposed to the CIE model show reductions during the acute withdrawal period followed by elevation during protracted abstinence (Vendruscolo et al., 2012). Finally, in postmortem brain tissue of individuals with an AUD, GR expression levels are reduced across several extrahypothalamic brain regions, including the prefrontal cortex, amygdala, and striatum (Gatta et al., 2021), as well as in the hippocampus (McClintick et al., 2013). These dynamic changes in GR expression may contribute to the elevated alcohol intake observed with chronic alcohol use and dependence. Indeed, pharmacological antagonism of GR has been shown to reduce alcohol intake in rodents, non-human primates, and humans, across a range of drinking paradigms and access periods (Koenig and Olive, 2004; Vendruscolo et al., 2012, 2015; Repunte-Canonigo et al., 2015; Jimenez et al., 2020; Savarese et al., 2020; Benvenuti et al., 2021; McGinn et al., 2021), suggesting that GR activation promotes alcohol intake. Genetic studies also support a role for GR in risk for AUD. Polymorphisms in the gene encoding GR, *NR3C1*, are associated with both age of onset of drinking and drinking to intoxication in adolescents, factors which are each strong predictors of developing an AUD (Desrivieres et al., 2011).

There are several extrahypothalamic brain regions that are likely important for glucocorticoids and GR, and one region where glucocorticoids may mediate the reinforcing effects of alcohol is the NAc (Spanagel et al., 2014). Glucocorticoids themselves exert positive reinforcing effects and potentiate dopamine release from the mesolimbic dopaminergic neurons (Piazza and Le Moal, 1997). Further, GR activation within the NAc enhances both appetitive and aversive learning (Wichmann et al., 2012), and administration of a GR antagonist directly into the NAc can decrease alcohol intake in alcohol-dependent rats (Repunte-Canonigo et al., 2015). Taken together, these data suggest the NAc is a critical area for the actions of glucocorticoids and GR in regulating alcohol intake.

Recently, we investigated the effects of GR antagonism on binge-like ethanol intake in the first replicate line of the High Drinking in the Dark (HDID-1) mice (Savarese et al., 2020). These mice were selectively bred for high blood alcohol levels (BALs) in a binge-like ethanol intake task, Drinking in the Dark (DID) (Crabbe et al., 2009), which utilizes a limited-access drinking paradigm during the circadian dark cycle and reliably produces intoxicating BALs (Rhodes et al., 2005). As such, HDID mice serve as unique genetic models of risk for binge-like ethanol intake. Because selective breeding principally changes the frequencies of genes affecting the targeted trait, any other differences between the selected line and its non-selected founder line may reflect coordinate genetic influences on the two traits. Thus, comparisons between HDID-1 and the founder HS/NPT lines have successfully been used to investigate the genetic and molecular determinants of high-risk drinking and identify potential pharmacotherapies for AUD (Cozzoli et al., 2012, 2016; Iancu et al., 2013, 2018; Crabbe et al., 2017, 2020; Ferguson et al., 2018, 2019; Grigsby et al., 2020; Ozburn et al., 2020; Pozhidayeva et al., 2020; Robinson et al., 2020; Savarese et al., 2020). To test the effects of GR antagonism within this selectively bred line, HDID-1 mice were given both mifepristone, a non-selective steroid hormone receptor antagonist, and CORT113176, a specific GR antagonist. Both compounds produced reductions in ethanol intake and BALs in DID in the HDID-1 mice (Savarese et al., 2020). Interestingly, the founder line of these mice, HS/NPT, did not show reductions in either ethanol intake or BALs in DID with GR antagonism, suggesting the selection process altered sensitivity to GR antagonism, perhaps through changes in either GR expression or activity.

Here, our goal was to begin to understand the mechanism driving increased sensitivity to GR antagonism in the HDID-1 mice by examining plasma corticosterone levels and mRNA expression of GR and its related chaperone proteins in the NAc of HDID-1 and HS/NPT mice. In Experiment 1 we evaluated plasma corticosterone levels at baseline or at varying timepoints (30, 60, or 120 min) after an injection of either saline or 2 g/kg ethanol, to capture stimulus-induced peak and recovery levels. We predicted that HDID-1 mice would have higher basal corticosterone levels than HS/NPT mice at baseline, suggestive of a dysregulation of HPA output after selection. In Experiment 2, we measured basal gene expression of GR (*Nr3c1*) in the NAc of HDID-1 and HS/NPT mice, as well as expression of two chaperone proteins of GR, FKBP51 (*Fkbp5*), and FKBP52 (*Fkbp4*). FKBP51 and FKBP52 are immunophilins that act in opposing ways to regulate GR transcriptional activity. When GR is bound to FKBP51, it is retained in the cytoplasm and prevented from translocating to the nucleus (Gray et al., 2017). When glucocorticoids rise and pass through the cell membrane to bind to GR, a conformational change leads to the shedding of FKBP51 and binding of FKBP52, which allows translocation into the nucleus. GR activation also leads to increased *Fkbp5* expression, resulting in negative feedback of GR transcriptional activity (Vermeer et al., 2003). The amounts of FKBP51 and FKBP52 in the cell can therefore have a profound effect on GR activity. In Experiment 2, we predicted elevated *Nr3c1* expression and/or reduced *Fkbp5* expression in the NAc of HDID-1 mice relative to HS/NPT mice, evidence of hyperactive GR activity in HDID-1 mice. In Experiment 3, we measured expression of these same genes in the NAc of HDID-1 and HS/NPT mice after either a saline or 2 g/kg ethanol injection. We predicted higher overall expression of *Nr3c1* and blunted *Fkbp5* expression after ethanol in HDID-1 mice, suggesting a dysregulation of the negative feedback loop that regulates GR activity.

## Materials and Methods

### Animals

Adult mice of both sexes of the HDID-1 line and of the genetically diverse founder stock, HS/NPT, were bred and maintained in the Veterans Affairs Portland Health Care System Veterinary Medical Unit (Crabbe et al., 2009, 2014). For age ranges and numbers of mice, see individual experiments below. Mice were bred and housed on a reverse light/dark schedule (12 L:12 D, lights off at 8:30 a.m.) and experimental rooms were maintained at a temperature of 21 ± 1°C. The light cycle remained unchanged for Experiments 2 and 3 (information on the light cycle for Experiment 1 is described below). Water and Purina 5 LOD chow (PMI Nutrition International, Brentwood, MO, United States) were provided *ad libitum*. Mice were housed in standard polycarbonate cages with stainless steel wire tops and Bed-o’cobs ^®^ bedding (The Andersons, Inc., Maumee, OH, United States). All procedures were approved by the local Institutional Animal Care and Use Committee of the VA Portland Health Care System and were conducted in accordance with NIH Guidelines for the Care and Use of Laboratory Animals.

### Experiment 1: Measurement of Plasma Corticosterone Levels in High Drinking in the Dark and HS/NPT Mice

**FIGURE 1:**
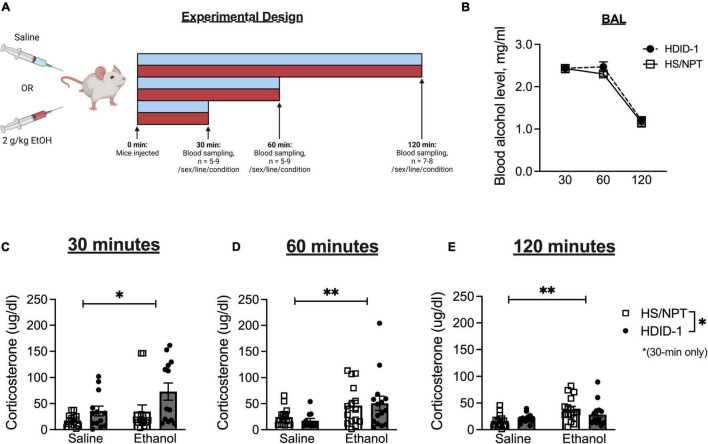
HDID-1 mice exhibit greater stimulated corticosterone response than HS/NPT founders. **(A)** Separate groups of HDID-1 and HS/NPT mice were given either a 2 g/kg ethanol injection or an equivalent volume of saline and blood samples were collected at either 30, 60, or 120 min after injections to measure corticosterone levels. **(B)** In mice that received ethanol injections, BALs were similar across time in the two genotypes. **(C)** At 30-min post-injection there was a significant effect of condition (ethanol > saline), genotype (HDID-1 > HS/NPT), and sex (females > males, data collapsed across sex in figure for ease of viewing). **(D,E)** At 60- and 120-min post-injection, the condition effect remained, but the sex and genotype effects were no longer evident. These data suggest that HDID-1 mice exhibit a greater stimulated response to injection. Means ± SE shown. ^∗^*p* < 0.05; ^∗∗^*p* < 0.01.

Two hundred and forty-nine HDID-1 and HS/NPT mice were used to measure corticosterone levels. Mice were 89–171 days of age and of generations S44.G48 (HDID-1) and G99.V31 (HS/NPT). At least 2 weeks prior to testing, mice were moved to a separate room and acclimated to a new light schedule (lights on at 1 p.m. and off at 1 a.m.). Mice remained group-housed with littermates throughout the experiment. Mice were pseudorandomized to condition and timepoint, and were split into five cohorts (*n* = 43–50/cohort) that were tested on separate days. Each combination of sex, genotype, condition, and timepoint were represented within a given cohort. A between-subjects design was employed for corticosterone measurements across timepoints in order to avoid sampling-induced stress responses (each animal was only sampled at one time). A schematic of experimental groups and timeline is shown in Figure 1A.

On the day of testing, mice were weighed at 12 p.m., 1 h before the lights on. Beginning 3 h into the light cycle, mice received either a 2 g/kg ethanol injection (20% v/v) or an equivalent volume of 0.9% saline (*n* = 5–9/line/sex/condition/timepoint). Either 30, 60, or 120 min following the injections blood samples were collected. Each mouse only provided a single blood sample. Sampling timepoints were designed to capture peak corticosterone levels (30 min), falling levels (60 min), and recovery (120 min) (Zgombick and Erwin, 1988). A separate group of mice that were being sampled for baseline corticosterone levels (*n* = 11–16/line/sex) received no injection and had blood samples collected 6 h into the light cycle. Blood samples were collected during the light cycle when corticosterone levels are low in rodents, to be able to detect stimulated corticosterone responses to ethanol.

To obtain tail bloods, mice were taken from their home cages and briefly restrained in Plexiglas restrainers. Tails were nicked approximately 2 mm from the tip, and 30 μl of blood was collected into heparinized capillary tubes which were then sealed with clay. Tail blood samples were kept on ice until testing was completed. Animals that received an ethanol injection had an additional periorbital blood sample taken immediately after the tail blood sampling for analysis of BALs. Periorbital blood samples were frozen at −20°C until gas chromatography was performed (Finn et al., 2007). All samples were taken within 2 min of cage disturbance.

Following testing, tail blood samples were centrifuged (3,000 rpm for 5–6 min) at 5°C to separate plasma. Plasma samples were then frozen at −20°C until radioimmunoassays were performed. Corticosterone concentrations were measured using a commercially available radioimmunoassay kit (ImmuChem Double Antibody Corticosterone for rodents, MP Biomedicals, Santa Ana, CA, United States), as described in Cozzoli et al. (2014). In brief, corticosterone concentration in plasma samples (5 μl) was single-determined via interpolation from a standard curve containing six standards that ranged from 25–1,000 ng/mL (i.e., 2.5–100 μg/dl).

### Experiment 2: Measurement of Baseline Gene Expression of Glucocorticoid Receptor and Chaperone Proteins in the Brains of High Drinking in the Dark and HS/NPT Mice

Forty-seven HDID-1 and HS/NPT mice (*n* = 11–12/line/sex) were euthanized for collection of whole brains. Mice were 69–80 days of age and of generations S41.G43 and S42.G44 (HDID-1) and G94.V26 (HS/NPT). Mice remained group-housed with littermates throughout the experiment. Because we were interested in measuring gene expression at the time of day that these mice typically consume ethanol, tissue collection began 7 h into the dark cycle to coincide with the end of a traditional 4-h DID binge-ethanol drinking session (Rhodes et al., 2005). Animals were removed from their home cage, cervically dislocated, and rapidly decapitated. Whole brains were removed and immediately put into dry ice for several minutes for rapid freezing. Brains were then stored at −80°C until tissue processing occurred.

### Experiment 3: Measurement of Stimulated Gene Expression of Glucocorticoid Receptor and Chaperone Proteins in the Brains of High Drinking in the Dark and HS/NPT Mice

98 HDID-1 and HS/NPT mice (*n* = 12–13/line/sex/condition) were injected intraperitoneally with either 2 g/kg ethanol or an equivalent volume of saline and were euthanized for collection of whole brains 4 h later. Mice were 60–105 days of age and of generations S43.G47 (HDID-1) and G98.V30 (HS/NPT). Because we were interested in measuring gene expression at the time of day that these mice typically consume ethanol, injections were administered in a staggered manner 2–4 h into the dark cycle to coincide with the start of a traditional DID binge-ethanol drinking session (Rhodes et al., 2005) and tissue collection coincided with the end of a 4-h DID session. One week prior to testing, mice were singly housed (as they would be during DID). On the day of collection, mice were weighed approximately 30 min prior to lights off. For brain collection, animals were removed from their home cage, cervically dislocated, and rapidly decapitated. Whole brains were removed and immediately put into dry ice for several minutes for rapid freezing. Brains were then stored at −80°C until tissue processing occurred.

### Quantitative Real-Time Polymerase Chain Reaction

Brains from Experiments 2 and 3 were processed in the same manner. Whole brains were mounted on a cryostat and sectioned into 200 μm-thick coronal sections at −18°C onto slides. The NAc was punched from sections using a 1 mm tissue puncher and tissue was placed into 1.5 mL centrifuge tubes. Tissue was stored at 80°C until it was sent to the Gene Profiling Shared Resource at Oregon Health & Science University for RNA isolation. For pre-processing, tissue samples were collected in 400 μl RLT Plus buffer and transferred to RB tubes (QIAGEN). All samples were mechanically disrupted with the TissueLyser at 30 Hz for 2 min. Lysates were then transferred to 2 mL Sarstedt screw-top tubes that were compatible with the QIAsymphony instrument. RNA was isolated using the QIAsymphony RNA Kit (QIAGEN) following the manufacturer’s recommended protocol and utilizing the QIAsymphony isolation robot, which includes an in-solution DNase step. RNA was eluted in 100 μl nuclease-free water. RNA concentration and yield were determined by UV absorption (using a NanoDrop 8000 spectrophotometer). RNA samples were then stored at −80°C and transferred back to our lab for cDNA synthesis. RNA (100 ng) was processed to cDNA using the BioRad iScript cDNA synthesis kit (Bio-Rad Laboratories, Inc., Hercules, CA, United States) according to the manufacturer’s protocol, as in Pozhidayeva et al. (2020). Quantitative real-time PCR was used to measure expression of GR (*Nr3c1*), FKBP51 (*Fkbp5*), FKBP52 (*Fkbp4*), and 18s (*Rps18*) using iQ Multiplex Powermix (Bio-Rad Laboratories, Inc.). The following PrimePCR™ Probe Assays were used (Bio-Rad Laboratories, Inc.): *Nr3c1* (Mouse, TEX 615, qMmuCIP0033656), *Fkbp5* (Mouse, Cy5, qMmuCEP0054766), *Fkbp4* (Mouse, HEX, qMmuCIP0028610), and *Rps18* (Mouse, FAM, qMmuCEP0053856).

### Data Analysis

The primary dependent variable in Experiment 1 was plasma corticosterone levels (μg/dl), at baseline and following intraperitoneal injections. A 2-way ANOVA (genotype × sex) was conducted to analyze baseline corticosterone levels. A 3-way ANOVA (genotype × sex × condition) was conducted within each timepoint to determine group differences in corticosterone levels following either a saline or ethanol injection. A 2-way ANOVA (genotype × timepoint) was conducted to evaluate group differences in blood ethanol levels across time.

For Experiments 2 and 3, the dependent variable of interest was normalized expression levels of each gene of interest. Normalized expression levels were calculated using a slightly modified version of the protocol published in Taylor et al. (2019). In brief, average quantification cycle (Cq) values in HS/NPT mice of both sexes (or in Experiment 3, HS/NPT mice that received saline) were obtained for each gene and were then used to calculate individual deltaCq scores (average Cq-individual Cq). Relative quantities (RQ) were then obtained for each individual gene (2^deltaCq) and 18s was used as a reference gene to calculate normalized expression (RQ of gene of interest/RQ of 18s). A 2-way ANOVA (genotype × sex) of normalized gene expression values was conducted for each gene of interest in Experiment 2. A 2-way ANOVA (genotype × sex × condition) of normalized gene expression values was conducted for each gene of interest in Experiment 3. Pairwise comparisons of interaction effects used a Sidak’s correction.

All statistical analyses, as well as the creation of all graphs, were completed in GraphPad Prism 9. BioRender was used to create illustrations of experimental schematics.

## Results

### High Drinking in the Dark Mice Exhibit Greater Stimulated Corticosterone Response Than Founders, HS/NPT

In order to investigate whether the selection process altered HPA axis output in the HDID-1 mice, we measured basal and stimulated blood plasma corticosterone levels in independent groups of adult HDID-1 and HS/NPT mice. In mice that received no injection (baseline samples), an overall sex effect was found [*F*(1, 49) = 4.692; *p* = 0.035], with female mice having greater corticosterone levels than male mice, but there was no effect of genotype or interaction (values in Table 1).

**TABLE 1:** Baseline corticosterone levels (μg/dl) in HDID-1 and HS/NPT mice.

	HDID-1	HS/NPT
Male	10.42 (3.70)	13.91 (2.40)
Female*	28.32 (8.39)	18.28 (3.79)
Overall (both sexes)	20.12 (5.11)	16.32 (2.35)

*Means (SEMs) shown.*

*Signifies a main effect of sex.

Separate groups of mice received intraperitoneal injections of either saline or ethanol, and plasma corticosterone levels were measured at different time points following injection in independent groups of mice (Figure 1A). In mice that received ethanol injections, BALs did not differ between genotypes [*F*(1, 81) = 0.943; *p* = 0.335], as seen in Figure 1B. In mice that had blood samples taken at the 30-min timepoint designed to capture peak corticosterone levels, a 3-way ANOVA revealed a significant main effect of condition [*F*(1, 48) = 5.288; *p* = 0.026; ethanol > saline], a main effect of sex [*F*(1, 48) = 20.80; *p* < 0.0001; female > male], and a main effect of genotype [*F*(1, 48) = 6.684; *p* = 0.013; HDID-1 > HS/NPT], but no significant interactions, as shown in Figure 1C (data is shown collapsed across sex for ease of viewing). At the 60-min timepoint that was designed to capture falling corticosterone levels, a 3-way ANOVA revealed a significant main effect of condition [*F*(1, 50) = 8.463; *p* = 0.005], with ethanol producing greater corticosterone levels than saline, but no other significant effects, as shown in Figure 1D. Finally, within mice that were sampled at the 120-min timepoint that was designed to sample recovery corticosterone levels, a 3-way ANOVA once again revealed a significant main effect of condition [*F*(1, 51) = 9.919; *p* = 0.003], with ethanol levels being greater than saline levels, but no other significant effects, as shown in Figure 1E.

Overall, we observed a greater stimulated corticosterone response from ethanol than saline, as expected. Our data also suggest that the selection process did not alter basal corticosterone levels in HDID-1 mice, but it did alter stress sensitivity, such that HDID-1 mice exhibited a greater peak corticosterone response to injection than the HS/NPT mice.

### High Drinking in the Dark Mice Have Reduced Basal *Nr3c1* and *Fkbp4* NAc Gene Expression Relative to Their Founders, HS/NPT

**FIGURE 2:**
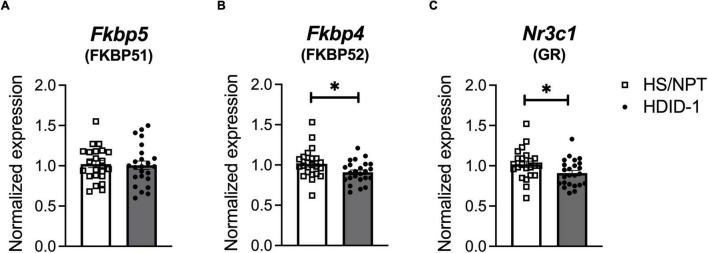
HDID-1 mice have reduced basal *Nr3c1* and *Fkbp4* NAc gene expression relative to their HS/NPT founders. Baseline gene expression of GR (*Nr3c1*), FKBP51 (*Fkbp5*), and FKBP52 (*Fkbp4*) was analyzed in the NAc of HDID-1 and HS/NPT mice (*n* = 11–12/sex/line). **(A)** No group differences were observed in *Fkbp5* expression. **(B,C)** HDID-1 mice have reduced expression of *Fkbp4* and *Nr3c1* relative to their founders, HS/NPT. Means ± SE shown. ^∗^*p* < 0.05.

Basal gene expression of GR (*Nr3c1*) and its chaperone proteins FKBP51 (*Fkbp5*) and FKBP52 (*Fkbp4*) were analyzed in the NAc of HDID-1 and HS/NPT mice (*n* = 11–12/line/sex) to determine whether selection may have altered GR expression or activity. A two-way ANOVA (sex × genotype) revealed no significant effects on *Fkbp5* expression (*p*’s > 0.078), as shown in Figure 2A. However, we observed reduced expression of both *Fkbp4* and *Nr3c1* in the NAc of HDID-1 mice relative to HS/NPT mice [*Fkbp4*: *F*(1, 43) = 5.560; *p* = 0.023; *Nr3c1*: *F*(1, 43) = 4.544; *p* = 0.039], as shown in Figures 2B,C. No other significant effects (i.e., sex or interaction) were observed (*p*’s > 0.110), so data are shown collapsed across sex. Together, these results suggest that selection for high BALs exerted changes in mRNA levels of GR and one of its chaperone proteins in the NAc of HDID-1 mice, such that these high drinking mice exhibit both reduced GR (*Nr3c1*) expression and reduced expression of *Fkbp4*, the gene encoding the chaperone protein responsible for translocating GR into the nucleus, relative to their founders.

### High Drinking in the Dark Mice Fail to Exhibit Ethanol-Induced Increase in *Fkbp5* Expression

**FIGURE 3:**
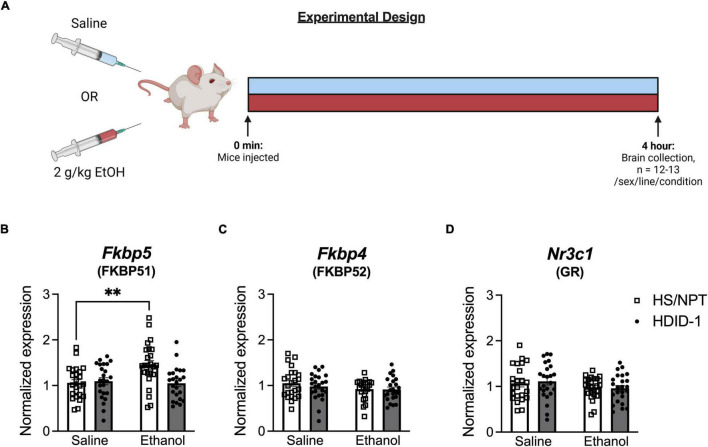
HDID-1 mice fail to exhibit ethanol-induced increase in *Fkbp5* expression. **(A)** Separate groups of HDID-1 and HS/NPT mice were given either a 2 g/kg ethanol injection or an equivalent volume of saline and brains were collected 4 h later for gene expression analysis in the NAc. **(B)** Ethanol injection produced an increase in *Fkbp5* expression in the HS/NPT mice that was not evident in HDID-1 mice. **(C,D)** No group differences were observed in either *Fkbp4* or *Nr3c1* expression. Means ± SE shown. ^∗^*p* < 0.01.

We next examined gene expression of *Nr3c1*, *Fkbp5*, and *Fkbp4* following ethanol exposure (via intraperitoneal injection of 2 g/kg ethanol vs. saline) in the NAc of HDID-1 and HS/NPT mice (Figure 3A). Here, analysis of *Fkbp5* expression resulted in a significant main effect of condition [*F*(1, 89) = 4.747; *p* = 0.032], genotype [*F*(1, 89) = 5.292; *p* = 0.024], sex [*F*(1, 89) = 4.542; *p* = 0.036], and a condition by genotype interaction [*F*(1, 89) = 7.620; *p* = 0.007]. Overall, ethanol induced greater *Fkbp5* expression compared to saline, male mice had greater expression of *Fkbp5* than female mice, and HS/NPT mice had greater *Fkbp5* expression than HDID-1 mice. Probing the condition by genotype interaction, however, revealed that ethanol induced an increase in *Fkbp5* expression in the HS/NPT mice (*p* = 0.002) that was not evident in HDID-1 mice (*p* = 0.903), as shown in Figure 3B. However, we observed no significant effects of group (genotype, sex, or condition) on gene expression of either *Fkbp4* or *Nr3c1* (*p*’s > 0.089), as shown in Figures 3C,D. Together, these data suggest that selection in HDID-1 mice blunted the ethanol-induced increase in *Fkbp5* expression observed in HS/NPT mice.

## Discussion

Glucocorticoids and GR have been shown to be important regulators of alcohol intake across species (Koenig and Olive, 2004; Junghanns et al., 2005; Vendruscolo et al., 2012, 2015; Repunte-Canonigo et al., 2015; Blaine et al., 2019; Jimenez et al., 2020; Savarese et al., 2020; Benvenuti et al., 2021; McGinn et al., 2021). Recently GR antagonism was shown to reduce ethanol intake in the selectively bred HDID-1 mice, but not in their founders HS/NPT, suggesting that selective breeding for high BALs enhanced sensitivity to GR antagonism, a finding that may have important implications for the genetic determinants of high-risk ethanol intake (Savarese et al., 2020). Here, we examined HPA axis responses and GR gene expression in the HDID-1 mice in order to begin to understand the mechanism underlying this enhanced sensitivity to GR antagonism. HDID-1 mice exhibited a higher peak corticosterone response to a mild stressor (intraperitoneal injection) than HS/NPT mice, which may suggest a more sensitive stress response in these mice. Contrary to our expectations, HDID-1 mice also had reduced basal mRNA expression of both GR (*Nr3c1*) and FKBP52 (*Fkbp4*) in the NAc relative to HS/NPT mice. Finally, while HS/NPT mice displayed an increase in *Fkbp5* expression after ethanol injection, HDID-1 mice did not. Together these data suggest that selection for genes leading to high BALs in the HDID-1 mice led to alterations in multiple aspects of the stress system, both peripherally (with circulating corticosterone levels) and centrally (with gene expression in the NAc). Given the reductions in binge-like drinking observed in HDID-1 mice with administration of GR antagonists (Savarese et al., 2020), these alterations in the physiological stress system may contribute to the elevated ethanol intake observed in these mice.

In Experiment 1, we examined circulating corticosterone levels at baseline and following a saline or ethanol injection in an effort to identify potential changes in HPA output that may have occurred as a result of the selection process. Individuals with an AUD have been shown to exhibit higher basal cortisol levels and a blunted cortisol response to stress, and this hyporesponsiveness of the HPA axis to stress is thought to drive relapse behavior (Adinoff et al., 2005; Junghanns et al., 2005; Blaine et al., 2019). Blunted cortisol responses to stress and alcohol cues can also be observed in individuals who engage in binge or heavy drinking relative to moderate drinkers, suggesting these changes in HPA output predate the development of an AUD (Blaine et al., 2019). Here, we observed no differences in basal corticosterone levels in animals that have a genetic risk for high ethanol intake compared to their lower-drinking founders. However, the HDID-1 mice had a greater stimulated corticosterone response than the HS/NPT mice, suggesting a greater sensitivity to stress. Although our results differ from those found in individuals after chronic alcohol use, they are similar to studies in individuals with a genetic risk for high alcohol intake. Male individuals with a positive family history of AUD were found to exhibit a heightened HPA reactivity [as measured by elevated cortisol as well as adrenocorticotrophin hormone (ACTH)] in response to a psychosocial stressor (the Trier Social Stress Test, TSST) relative to family history negative subjects (Zimmermann et al., 2004). Elevated cortisol levels in response to the TSST were also observed in Caucasian subjects with an AUD-positive family history relative to controls in a separate study (Uhart et al., 2006). These data would suggest that genetic risk factors for high alcohol use linked to HPA output are distinct from the changes observed in HPA output as a consequence of alcohol use, where a blunted response to stress can be observed. Further, high cortisol reactivity has also been associated with increased alcohol consumption. In rhesus macaques that underwent a series of stressful social separation challenges, subjects that responded to these stressors with high cortisol levels consumed more alcohol in subsequent access periods than subjects that had low cortisol responses (Fahlke et al., 2000). Genetic predisposition to high alcohol intake may interact with cortisol output to drive alcohol intake. Individuals with a positive family history of AUD who were high cortisol responders to the TSST drank more alcohol than control subjects who were high cortisol responders (Brkic et al., 2015). Interestingly, although binge/heavy drinkers exhibit blunted cortisol responses to alcohol cues relative to moderate drinkers, they show a greater cortisol response to alcohol itself in the 30-min following consumption (Blaine et al., 2019). The discrepancy in cortisol responses of high-risk drinkers to different stimuli suggests that dysregulation of HPA output with alcohol use is not necessarily a linear process.

Glucocorticoids are not only released in response to stress or alcohol exposure, but are also dynamically released throughout the circadian cycle (Gray et al., 2017). Abstinent individuals with an AUD demonstrate blunted cortisol awakening responses and decreased cortisol pulse amplitude at night compared to age-similar controls (Adinoff et al., 2005). Further, total cortisol release correlated inversely with craving in individuals with a positive family history of AUD (Adinoff et al., 2005), supporting a role for low cortisol levels in promoting alcohol intake. These data might suggest that HDID-1 mice would have reduced total corticosterone output across the circadian cycle. While we did not observe any differences between the genotypes in basal corticosterone levels, one limitation of our study design is that we only sampled corticosterone levels during a small portion of the day, and were therefore unable to track circadian and ultradian fluctuations. Blood sampling occurred during the light cycle when corticosterone levels are lower in rodents, in order to be able to observe stimulated corticosterone responses with ethanol exposure. It remains unknown whether HDID-1 mice have similar basal levels during the dark cycle (rodents’ active phase) and total corticosterone output across the circadian cycle compared to HS/NPT mice, or whether the selection process may have altered circadian or ultradian rhythms of corticosterone as well.

It is also important to note that corticosterone levels in the blood do not always correlate with brain corticosterone levels. Chronic alcohol consumption followed by a withdrawal period can lead to elevated brain glucocorticoid levels in rodents that are region-dependent, and these changes are not mirrored in plasma concentrations (Little et al., 2008). However, the withdrawal period was necessary to induce changes in brain glucocorticoid levels, as continued alcohol exposure did not result in these regional differences, nor did short-term alcohol consumption followed by abstinence. Given the experiment conducted here with HDID-1 and HS/NPT mice evaluated corticosterone levels in the blood after a single acute exposure to 2 g/kg alcohol, there is no evidence to suggest that these corticosterone levels would not be representative of brain glucocorticoid levels. Nevertheless, because we do not fully understand how the selection process may have altered the glucocorticoid system in the HDID-1 mice, it is possible that the corticosterone levels we measured in blood could be distinct from regional brain concentrations.

HPA activation is regulated by an extensive extrahypothalamic network, including limbic and prefrontal cortical inputs to the hypothalamus. It is within these extrahypothalamic brain regions that chronic alcohol use is thought to sensitize GR signaling (Edwards et al., 2015). Male Wistar rats made dependent on alcohol via CIE vapor exposure display reductions of GR mRNA during the acute withdrawal period and elevations in GR mRNA during protracted abstinence in several of these extrahypothalamic stress- and reward-related brain regions, including the NAc (Vendruscolo et al., 2012). Further, GR antagonism, delivered directly into the NAc, can reduce the elevated alcohol consumption observed in dependent rats (Repunte-Canonigo et al., 2015). Because the role of GR in the NAc seemed to be particularly important for regulating alcohol intake, in Experiments 2 and 3 we sought to examine whether the selection process altered expression of GR and its regulator proteins in the NAc of HDID-1 mice.

Experiment 2 evaluated baseline gene expression in the HDID-1 mice and their founders, HS/NPT. Reduced expression of *Nr3c1* (GR) and *Fkbp4* (FKBP52) were observed in the HDID-1 mice. Given the sensitivity of HDID-1 mice to GR antagonists, we had hypothesized that HDID-1 mice would have elevated GR expression or activity (which could be inferred from reduced *Fkbp5* expression). Interestingly, although GR antagonism has been shown to effectively reduce drinking during the protracted abstinence period when expression of GR is elevated, the same antagonist can also be administered prior to the onset of alcohol dependence and prevent escalated intake, when presumably no changes in GR expression have yet taken place (Vendruscolo et al., 2012). This would suggest that the effectiveness of GR antagonism in reducing alcohol intake is not dependent on enhanced GR expression. Indeed, GR antagonism may be effective at reducing alcohol intake when GR is actually reduced in expression. Mifepristone administration in non-treatment seeking individuals with AUD reduced both alcohol craving and consumption (Vendruscolo et al., 2015), and evidence from postmortem tissue of individuals with AUD without protracted abstinence from alcohol suggests reduced *Nr3c1* (GR) expression throughout the brain would be evident (McClintick et al., 2013; Gatta et al., 2021). Because glucocorticoids are released with alcohol exposure, decreased GR expression might be expected after chronic alcohol use. Repeated stress or corticosterone administration has been shown to reduce the number of glucocorticoid receptors in the rat brain in a region-specific manner, with reductions found in extrahypothalamic brain regions but not in the hypothalamus or pituitary (Sapolsky et al., 1984). There is also evidence that decreased GR expression in the NAc could increase risk for elevated alcohol intake, even without a history of alcohol use. Increased alcohol consumption and motivation was observed in mice that had experienced early life stress (maternal separation) and exhibited reduced expression of *Nr3c1* in the NAc (Garcia-Gutierrez et al., 2016).

In Experiment 3, we evaluated expression of these same genes after ethanol injection, at a time of day when these mice are known to consume enough alcohol to become intoxicated. Here, we found no significant effects for either *Nr3c1* or *Fkbp4* expression, but we observed a significant increase in *Fkbp5* expression in the HS/NPT mice after ethanol exposure that was not evident in the HDID-1 mice. *Fkbp5* expression increases with glucocorticoid receptor activation, an effect that helps to regulate GR transcriptional activity (Vermeer et al., 2003; Gray et al., 2017). Acute ethanol exposure induces glucocorticoid release and has also been shown to induce *Fkbp5* expression in mice (Treadwell and Singh, 2004). The failure of HDID-1 mice to exhibit this ethanol-induced increase in *Fkbp5* expression, particularly when the same dose of ethanol produced a robust glucocorticoid response in these mice (in Experiment 1), might suggest a maladaptive response to ethanol in the HDID-1 mice which leads to prolonged activation of GR transcriptional activity (without elevations in FKBP51 to inhibit GR) and/or a shifting in transcriptional targets in these mice away from *Fkbp5*, at least in the NAc. Interestingly, although administration of an FKBP51 (*Fkbp5*) inhibitor, SAFit2, reduced alcohol consumption in a two-bottle choice test in mice (Konig et al., 2020), the same inhibitor was ineffective at reducing alcohol intake in the HDID-1 mice in DID (Savarese et al., 2020). Although two-bottle choice and DID drinking are distinct, and it is still unknown whether SAFit2 would reduce two-bottle choice alcohol intake in the HDID-1 mice, it may be that the lack of ethanol-induced *Fkbp5* expression in the HDID-1 mice contributed to the failure of SAFit2 to reduce binge-like ethanol intake in these mice. However, it is important to acknowledge that the effects on gene expression of an ethanol injection (as in Experiment 3) may be quite distinct from those that would be observed with ingestion of ethanol (as in DID). We chose to examine gene expression after an ethanol injection rather than DID to ensure that the dose of ethanol would not vary between mice, as the two lines differ drastically in amount of ethanol consumed in DID. Nonetheless, it is possible that drinking ethanol may induce different gene expression responses in these mice than that of an ethanol injection. Further, thousands of genes are regulated by GR, and gene targets can be dependent on cell type, GR isoform, posttranslational modifications of GR, and dimerization patterns of GR (Oakley and Cidlowski, 2011). It may be that selection for high BALs modified GR protein levels and/or functioning in a manner we were unable to capture in this study design by examining mRNA expression.

The experiments herein were designed to explore whether there were differences in aspects of the stress system involved in the pharmacological actions of GR antagonists that may suggest a mechanism for the increased sensitivity of HDID-1 mice to GR antagonism. The results suggest that selection for high BALs led to both peripheral and brain changes in the physiological stress response system that may contribute to the elevated binge-like consumption in HDID-1 mice, but much remains unknown. Although our results demonstrated differences in mRNA expression of GR and the immunophilins FKBP51 and FKBP52, it is not known whether these translate to differences in protein levels. Further, GR has two primary isoforms, GRα and GRβ, that can have opposing effects on transcriptional activity (Oakley and Cidlowski, 2011). A history of ethanol intake can alter GR isoform expression within stress- and reward-related circuitry, and these changes can occur prior to the adaptive HPA changes observed with chronic ethanol consumption (Alhaddad et al., 2020). It is possible that the GR isoform expression of HDID-1 mice differs from that of HS/NPT mice and contributes to the elevated ethanol intake in HDID-1 mice. Alternatively, there may be differences in the cellular localization patterns of GR in the HDID-1 mice. Although GR is traditionally thought of as a transcription factor, it can also localize at the cellular membrane where it signals via rapid, non-genomic mechanisms (Oakley and Cidlowski, 2013). Membrane-bound GR can induce signaling cascades that culminate in transcription of genes distinct from that of cytoplasmic GR, adding to the complexity of factors involved in GR signaling (Rainville et al., 2019). Interestingly, the effect of GR antagonism on ethanol intake in HDID-1 mice occurred more rapidly than might be expected if the actions were due to genomic signaling of GR (2-h after drug administration) (Savarese et al., 2020), suggesting that the GR antagonist may have been acting on membrane-bound GR in the HDID-1 mice. It is possible that the lack of effect in HS/NPT mice vs. HDID-1 mice observed with GR antagonism was due to differences in GR localization within the cell and therefore differences in the downstream effects of drug administration.

We chose to evaluate expression levels of GR and the immunophilins FKBP51 and FKBP52 in these experiments, but there are many other proteins that regulate GR activity within the cell. Heat shock protein 90 (Hsp90) is another chaperone protein of GR which helps to bind glucocorticoids to GR (Kaziales et al., 2020), and two Hsp90 inhibitors (geldanamycin and alvespimycin) recently emerged as candidate drugs predicted to reduce alcohol consumption in HDID-1 mice using a transcriptome-based drug discovery paradigm (Ferguson et al., 2018). Another potentially relevant protein involved in GR regulation is the mineralocorticoid receptor (MR), which glucocorticoids preferentially bind at low levels and can heterodimerize with GR and influence downstream transcriptional targets (Gray et al., 2017). Levels of aldosterone, a ligand for MR, increase with chronic alcohol exposure in monkeys and are associated with craving and alcohol intake in alcohol-dependent patients (Aoun et al., 2018). Antagonizing MR also effectively reduced alcohol self-administration when administered systemically or directly into the central amygdala (CeA) of rats (Makhijani et al., 2020). Steroid receptor co-activator 1 (SRC-1), a histone acetyltransferase, also regulates GR activity and may be an important epigenetic factor in the response of GR to alcohol (Edwards et al., 2015). Finally, 11β-hydroxysteroid dehydrogenases (11β-HSD) convert glucocorticoids into active and inactive forms and are therefore integral to the downstream effects of glucocorticoids. Carbenoxolone, a non-selective 11β-HSD inhibitor reduced alcohol intake in male mice and rats in multiple drinking paradigms (Sanna et al., 2016). Expression levels of any of these GR-related proteins could be altered in the HDID-1 mice and contribute to alcohol intake.

We also chose to only evaluate gene expression within a single brain region, the NAc. Although the reasoning for exploring GR activity within the NAc was sound, there are multiple brain regions that have been implicated in GR-induced alcohol intake that would be reasonable targets for future study. Administering a GR antagonist directly into the CeA was sufficient to reduce alcohol consumption in dependent rats, suggesting a critical role for GR on alcohol intake in this brain area (Vendruscolo et al., 2015). Similarly, GR levels change with chronic ethanol exposure in the mPFC in both rats and humans (Somkuwar et al., 2017; Gatta et al., 2021), and may contribute to the transition to problematic alcohol intake (Lu and Richardson, 2014).

Ideally, future studies would examine additional brain regions in the HDID-1 mice and begin to address whether HDID-1 mice have altered expression of additional GR-related proteins or differences in GR isoforms or cellular localization. However, the HDID-1 mice are no longer being selectively bred. Instead, the HDID-1 mice were recently inbred to create the iHDID-1 line, a more genetically stable mouse line that still captures the unique genetic risk profile of the HDID-1 mice. These iHDID-1 mice have begun to be characterized and have retained the attenuated ethanol conditioned taste aversion that was a persistent phenotype in the HDID-1 mice (Crabbe et al., 2019). The iHDID-1 mice will be integral for future studies expanding on the findings here.

Taken together, our results herein suggest a potential physiological mechanism underlying the increased sensitivity to GR antagonism exhibited by the selectively bred HDID-1 mice. Selection for high BALs after a binge-drinking session in these mice was associated with alterations in HPA responsivity and gene expression of GR and its chaperone immunophilins in the NAc relative to the founder line, HS/NPT. Although future studies are needed to determine how these changes are associated with the elevated ethanol intake of HDID-1 mice, they suggest that the physiological stress system is an important genetic determinant of high-risk binge-like ethanol intake.

## Data Availability Statement

The raw data supporting the conclusions of this article will be made available by the authors, without undue reservation.

## Ethics Statement

The animal study was reviewed and approved by the Institutional Animal Care and Use Committee of the VA Portland Health Care System.

## Author Contributions

AS, JC, and AO were responsible for overall study concept and design. DF advised on the design of Experiment 1 and her team processed corticosterone data. AS, KG, BJ, and MB were responsible for the acquisition of all data. AS, KG, and BJ processed tissue, plasma, and blood samples. AS analyzed data and interpreted data outcomes with assistance from AO. BJ created the experimental design schematics for figures. AS drafted the manuscript and prepared figures with contributions from AO and JC. All authors provided feedback on the manuscript and approved the final version for publication.

## Conflict of Interest

The authors declare that the research was conducted in the absence of any commercial or financial relationships that could be construed as a potential conflict of interest.

## Publisher’s Note

All claims expressed in this article are solely those of the authors and do not necessarily represent those of their affiliated organizations, or those of the publisher, the editors and the reviewers. Any product that may be evaluated in this article, or claim that may be made by its manufacturer, is not guaranteed or endorsed by the publisher.
